# Wolves, but not dogs, are prosocial in a touch screen task

**DOI:** 10.1371/journal.pone.0215444

**Published:** 2019-05-01

**Authors:** Rachel Dale, Sylvain Palma-Jacinto, Sarah Marshall-Pescini, Friederike Range

**Affiliations:** 1 Wolf Science Center, Domestication Lab, Konrad-Lorenz Institute of Ethology, University of Veterinary Medicine, Vienna, Austria; 2 Comparative Cognition, Messerli Research Institute, University of Veterinary Medicine, Vienna, Austria; 3 University of Tours, Parc Grandmont, Tours, France; Consiglio Nazionale delle Ricerche, ITALY

## Abstract

Prosociality is important for initiating cooperation. Interestingly, while wolves rely heavily on cooperation, dogs’ do so substantially less thus leading to the prediction that wolves are more prosocial than dogs. However, domestication hypotheses suggest dogs have been selected for higher cooperation, leading to the opposing prediction- increased prosocial tendencies in dogs. To tease apart these hypotheses we adapted a paradigm previously used with pet dogs to directly compare dogs and wolves. In a prosocial choice task, wolves acted prosocially to in-group partners; providing significantly more food to a pack-member compared to a control where the partner had no access to the food. Dogs did not. Additionally, wolves did not show a prosocial response to non-pack members, in line with previous research that social relationships are important for prosociality. In sum, when kept in the same conditions, wolves are more prosocial than their domestic counterpart, further supporting suggestions that reliance on cooperation is a driving force for prosocial attitudes.

## Introduction

Humans show wide-ranging and regular prosociality (i.e. a voluntary action which benefits another[1]) [2,3], however phylogenetic relatedness to humans does not seem to explain the extent of prosociality other species show [4–6]. While chimpanzees show prosocial responses in some tasks [7–9], in many they do not [4,10–12], whereas some more distantly related monkey species show more consistent prosociality [6,13–17]. Therefore, recent studies have taken more of a comparative approach to consider possible convergent selection pressures that may be at play. In line with this approach, pet dogs have been considered as a good model as they are not closely related to humans but share the same environment [18]. Indeed it has recently been found that pet dogs do show prosociality towards familiar conspecific partners in a simplified prosocial choice task, where they could choose for how many trials to continue delivering a reward to a partner [19,20]. Some domestication hypotheses suggest that dogs have been selected for higher tolerance and cooperation [21], thus predicting that dogs should show prosocial tendencies. Therefore these findings may suggest that prosocial tendencies could have appeared during the course of domestication, meaning that their social proximity to humans drove the evolution of more prosocial behaviour.

Alternatively, a second possibility is that prosocial behaviour is a derived trait from dogs’ ancestors. Wolves are the closest living relatives of dogs and are therefore an important comparative model to tease these alternatives apart. Interestingly, wolves, like humans, cooperate extensively, both in captivity and in the wild [22,23] for many aspects of their life, including breeding [24], hunting [25] and territory defence [26]. Therefore, in addition to assessing the effects of domestication on dog prosociality, wolves make an excellent model for testing recent convergent evolution hypotheses. In particular, it has been suggested that prosociality is an important behaviour for initiating cooperation in both human and non-human species [6,27,28] and more specifically some findings posit that the extent of cooperation in breeding and raising young is a principally important explanatory variable for species differences in primate prosociality [6], recently also supported in birds [28]. Thus, based on the cooperative nature of wolf society, the ‘socio-ecology’ [29] and ‘cooperative breeding’ [6] hypotheses would predict that wolves should be equally, if not more, prosocial than dogs. Therefore we aimed at testing whether wolves (N = 9) would also be prosocial in a similar task to that employed with pet dogs. Additionally, in order to allow a direct comparison with dogs, we also tested identically kept pack-living dogs (N = 6) on the same task.

Furthermore, the studies with pet dogs revealed that they were prosocial to familiar individuals while withholding rewards towards strangers in comparison to control conditions where no partner received the reward [19,20]. Since dogs form social bonds with group members [30], but defend their territory against dogs of other neighbouring groups [31], group membership could explain the contrasting behaviour to familiar and stranger partners. Alternatively, dogs might just base their prosocial behaviour on familiarity (knowing the other individual or not) rather than group membership. Therefore we also tested the wolves and dogs of the current study with partners from the same pack (in-group) as well as well-known individuals from a different pack (out-group). If group membership, rather than mere familiarity, affects prosocial tendencies, we would expect both species to deliver more food to pack members than non-pack members.

**Fig 1:**
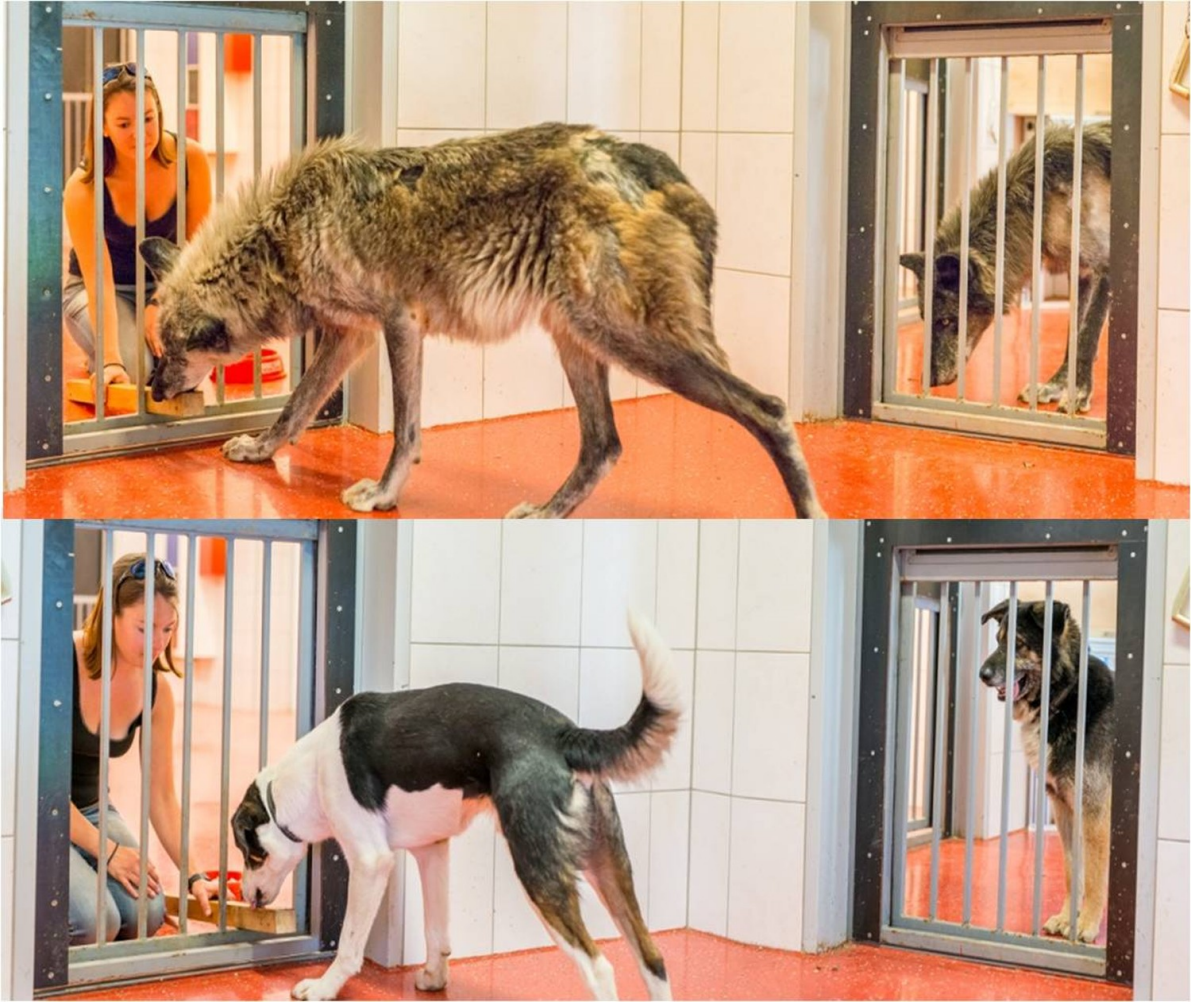
Set-up of the test condition. After a ‘giving’ choice by the subject (right), the partner (left) was rewarded. A transparent door between the two enclosures allowed the subject to see whether a partner was rewarded for their actions or not, as well as the identity of the partner. See also S1 Fig.

In this task the animals were trained to choose a ‘giving’ symbol, which delivered a reward to an adjacent receiver enclosure, over a ‘control’ symbol which provided no reward, by pressing it with their nose on a touch screen. We chose this 0/1 vs. 0/0 method (i.e. the subject can choose between rewarding only the partner or no reward at all) rather than the 0/1 vs. 1/1 method (a choice between rewarding only the self or both the self and the partner) because in our work with pet dogs it was established that the latter paradigm is too complex for dogs to learn (see [32]), as has also been found in other species [33]. During the training the subjects had access to the receiver enclosure and could thus access the rewards after a ‘giving’ choice. Subsequently, in the test and control conditions the transparent door between the enclosures was closed, therefore preventing access to the reward by the subject (Fig 1). In the test condition the partner was in the receiver enclosure and had access to any rewards delivered by the subject. In the control condition the partner was present, but in a different enclosure where they no longer had access to the reward (S1 Fig). This condition controls for the influence of social facilitation, where the mere presence of a conspecific can increase, or inhibit, an individual’s motivational state, and in turn this can enhance or decrease its engagement in certain behaviours [34]. Although this social facilitation control is considered to be the better control, since only one factor is changed in the set-up at the given time (whether the partner receives food or not) and despite the fact that, if employed, it has been shown to be rather important [19,35], few studies have used it so far [36]. Finally, in line with previous studies, we also included a non-social control where no partner was present at all.

Because the test occurred under extinction conditions, in that the subject was not rewarded during test/control sessions, *motivation sessions* were run between each condition in order to ensure that the motivation to work on the task was similar prior to each condition. These sessions were identical to the training sessions, whereby the subject had access to the receiver enclosure, allowing them to retrieve rewards. Furthermore, it is possible that subjects cease to work in the test/control due to stress, distraction or lack of motivation on that day. Therefore, immediately at the end of each test/control session the subject and partner remained in their original locations and the subject was presented with six *knowledge-probe trials*, where touching the giving symbol now delivered food to its enclosure.

Based on previous results with pet dogs, we predicted the pack dogs in the current task would show a prosocial response. There are two possible predictions for the wolves’ performance relative to the dogs’: 1) if prosociality has emerged during the course of domestication, wolves should be less prosocial than dogs, 2) if reliance on cooperation is a driver of prosocial regard, wolves should be at least as, if not more, prosocial than dogs.

## Results

A generalized linear mixed model (GLMM) revealed an interaction between species and condition on the number of trials in which the giving symbol was chosen (χ^2^ = 13.53, df = 4, p = 0.009), therefore each species was considered separately.

### Wolves

**Fig 2:**
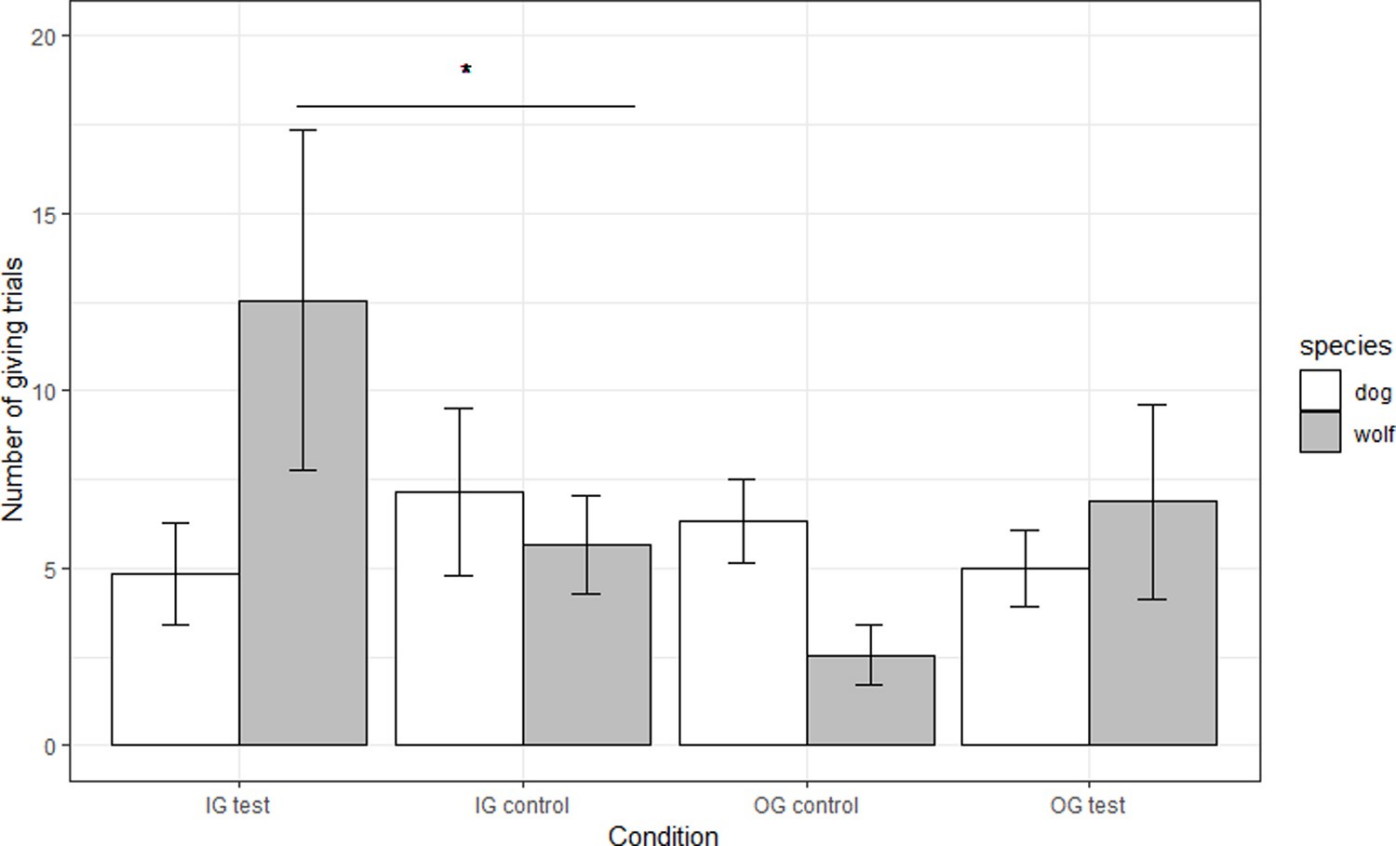
Number of giving trials performed by wolves and dogs in the test and respective social facilitation control conditions. IG = in-group, OG = out-group. Test = partner had access to the reward, control = partner was in present but had no access to the reward. Error bars represent standard error of the mean. The values depict the raw data.

Wolves showed no effect of session (χ^2^ = 6.19, df = 4, p = 0.19) indicating the frequency of delivering food to the adjacent enclosure did not change over time. An effect of condition emerged (χ^2^ = 13.94, df = 4, p = 0.007). Firstly, there was no difference between the three control conditions (in-group social facilitation (IG-control), out-group social facilitation (OG-control) and non-social control; χ^2^ = 4.79, df = 2, p = 0.09, S4 Fig). Secondly, wolves performed significantly more trials when the in-group partner could receive the reward (IG-test) than when the partner was present but unable to get the reward (IG-control; Z = -2.02, p = 0.04, Fig 2). There was no such effect of condition with the out-group partner (OG-test vs OG-control, Z = -1.76, p = 0.08).

### Dogs

Contrary to the results in the wolves, in the dogs there was an effect of session (χ^2^ = 22.07, df = 4, p = 0.0002) with performance decreasing across sessions, but no effect of condition emerged (χ^2^ = 1.37, df = 4, p = 0.85) indicating that the pack-dogs did not perform differently according to whether it was test vs. control or an in-group vs. out-group partner.

Importantly, every subject of both species performed all six knowledge-probe trials at the end of each session. In these trials, both subject and partner remained in their original location and the subject could receive food for themselves. This demonstrates that the subjects did not stop working on the touch screen due to distraction or stress from the presence of a partner, and they were willing to resume working immediately when they could receive the rewards themselves.

## Discussion

In a directly comparable set-up, we detected prosocial responses in wolves, but not dogs, in an experimental prosocial choice task. Our results support the predictions of the ‘socio-ecology’[29] and ‘cooperative breeding’ hypotheses [6], that the reliance on cooperation in wolves has shaped other aspects of their behaviour, including prosocial regard. The fact that wolves, but not dogs, were prosocial in the same task corroborates other findings that wolves are more tolerant with food sharing, a naturalistic measure of prosociality, than dogs [37]. Furthermore, wolves have been shown to be far more successful in experimental cooperation tasks than dogs [22], and are more sensitive to unequal reward distributions [38]. Wolves extensively rely on cooperation for many aspects of their lives including breeding [24], hunting [25] and territory defence [26]. Dogs, on the other hand, cooperate less than wolves in free-ranging settings, usually foraging solitarily and raising offspring alone [29]. The current study therefore further strengthens the suggestion that prosocial behaviour is an important factor for cooperative species, and taken together these experiments support a broader link between cooperation, prosociality and inequity aversion [6,39,40].

It is notable that, although the wolves were prosocial towards in-group pack members, they did not show prosocial regard to out-group partners. This result cannot be explained by kin selection as the most prosocial individuals (Aragorn, Wamblee and Tala) were not related to their in-group partners. In some species the behaviour of the partner can explain the prosocial response of the donor (e.g. [41]), however this has not been observed for dogs [20]. Since the current study was designed to compare wolves and dogs in a controlled manner, the current set-up offered limited behavioural options for the partner to influence the subject’s behaviour. Therefore, we do not expect partner behaviour to have greatly affected the response of the donors, but future research is necessary to fully test this.

Overall this positive response toward pack-members is in line with results in other species, that the social relationship is important for animals in deciding whether to act prosocially or not [19,42,43]. Furthermore, pet dogs were not prosocial towards strangers; in fact they delivered more food to an empty enclosure than to a stranger. However, in those studies we were unable to tease apart whether this was due to familiarity (i.e. knowing the partner at all) or group membership (knowing the partner, but belonging to a different group). Interestingly, in the current task wolves did not perform more trials for the out-group partner than in the control, but they also did not perform significantly less, as the pet dogs did. Since in-group members are more familiar than out-group members, the relative importance of familiarity versus group membership cannot be disentangled, but the fact that out-group members did not receive prosocial donations demonstrates that group-membership had an effect on donor behaviour. Although caution must be taken when comparing the pet dogs with the current experimental groups, this may suggest a gradient of prosocial regard, depending on the relationship with the partner (stranger–out-group–in-group).

In the current task dogs did not show any prosocial responses towards their partner. These results cannot be explained by a lack of understanding of the task [44] as wolves and dogs did not show a difference in how many sessions it took to reach criteria in the training (see S1 Data) and all dogs completed at least one trial, with most completing more than five. Furthermore, they reached criteria again in the intermittent motivation sessions, where the subjects had access to the reward location. Additionally the dogs always resumed working on the touch screen during the knowledge-probe trials at the end of each session, where the rewards were delivered to the subject’s enclosure, suggesting they were willing to work in these conditions when they themselves received the food. Furthermore, although there is a difference in sample size between wolves and dogs, this is unlikely to have fully explained the different results as only three dogs performed more trials in the in-group test than in the control, and the highest difference score was six trials, compared to 23 trials in the wolves. Indeed, since the number of trials performed decreased across test/control sessions, but not in the intermittent motivation sessions, it seems that as long as the dogs were not receiving food for themselves, they simply lost motivation in the task.

Subtle changes in methods can affect results in prosocial experiments, even within the same species [19,45], so we need to be cautious about directly comparing the current results with those from pet dogs, where prosociality has been observed. However, it is possible that socialisation, whereby pet dogs are ‘trained’ or encouraged to be more tolerant towards other dogs, or punished for ‘antisocial’ behaviour, results in higher prosociality than pack-living dogs that have been raised with no direct intervention by humans on their behaviour towards conspecifics.

Teasing apart the effects of socialisation on dog social cognition will be an interesting avenue to pursue. Regardless of what future research may reveal about the role of socialisation and/or methodology on prosociality in different dog populations, the important aspect of the current study is that in a directly comparable task with similarly raised and kept populations, wolves are more prosocial than dogs towards conspecific partners. We suggest this can be explained by the different socio-ecological selection pressures faced by the two species [29], with wolves having a higher reliance on cooperation with group members than their domestic counterpart. These results suggest that prosocial behaviours that have been shown to be present in pet dog populations are likely to be derived from ancestral traits [46].

## Methods

### Subject details

All subjects (N = 15, see Table 1) were hand-raised in peer groups at the Wolf Science Center (WSC) in Ernstbrunn, Austria, after being separated from their mothers in their first ten days. They were bottle-fed and later hand-fed by humans and had continuous access to humans in the first five months of their life. After five months, they were introduced into the packs of adult animals and currently live in large 2000-8000m^2^ enclosures. All animals receive training or partake in tests on a daily basis. For the current experiment, adult wolves (N = 9) and dogs (N = 6) were tested with a pack member and a relatively unfamiliar, non-pack member partner.

Individuals from other packs at the Wolf Science Center were used as the non-pack member partners and can be considered as out-group members. Although many of the pairs had lived together previously and often saw each other during routine movement of the animals, none had lived together for at least two years (except for Nuru who lived with his out-group partner 1yr 8months previously), compared with the in-group pack member partners that they had been living with for at least two years (with the exception of Yukon who had been living with her brother for 1yr 5months). Within subjects the in-group and out-group partners were the same sex, with the exception of Kenai who showed overt aggression to all other males. In order to avoid reciprocity effects, none of the dyads were tested in the reverse roles.

**Table 1:** Subject details.

Subject	Subject Sex	In-group Partner	Out-group Partner	Partner Sex
Wolves				
Kaspar	M	Shima	Yukon	F
Aragorn	M	Tala	Yukon	F
Shima	F	Aragorn	Nanuk	M
Yukon	F	Geronimo	Amarok	M
Kenai	M	Amarok	Yukon	M/F
Wamblee	M	Yukon	Tala	F
Tala	F	Chitto	Amarok	M
Ela	F	Etu	Wamblee	M
Etu	M	Maikan	Wamblee	M
Dogs				
Asali	M	Bora	Nia	F
Enzi	M	Zuri	Bora	F
Meru	M	Hiari	Banzai	M
Nuru	M	Enzi	Banzai	M
Pepeo	M	Nuru	Sahibu	M
Zuri	F	Pepeo	Asali	M

### Experimental setup

A mechanical touch screen (S2 Fig), built and designed at the Comparative Cognition Unit, Messerli Research Institute of the University of Veterinary Medicine, Vienna, was used to present the choices and subjects could select the chosen outcome by pressing their nose against one stimulus or the other. The touch screen was chosen as it was similar to the token choice task previously employed, which used wooden tokens, but we identified some additional advantages of the touch screen. Namely, the animals were already familiar with using, and making discriminations on, the touch screen and the automated set-up prevents potential human handling biases.

As the touch screen was permanently fixed, the subject was placed in the touch screen room while the partner was in an adjacent room (S1 Fig). The animals were separated by metal bars and plexi-glass so they could have visual, olfactory and auditory contact but could not come into direct physical contact.

All subjects were already familiar with using, and making discriminations on the touch screen. For this experiment, each subject was randomly assigned two stimuli (a ‘giving’ and a ‘control’ symbol) from a pool of three stimuli (S3 Fig). The stimuli were presented together on semi-randomized sides such that one stimulus did not appear on the same side more than twice in a row. For each session, the touch screen program automatically recorded the number of trials the subject participated in, as well as the number of trials for which the subject chose the 'giving’ symbol and the latency to choose a symbol. While all animals were previously touch screen trained, they were naïve in relation to the posed questions and symbols. So far they have been tested on non-social tasks such as numerical competence and learning by exclusion studies.

### Training

Subjects were trained to choose the giving symbol using positive reinforcement. When the subject chose the giving symbol, by pressing it with their nose, a clicker was used by a helper standing outside to signal a correct choice and the subject was rewarded by the experimenter, who slid a stick with a piece of sausage on it into the partner room (Fig 1). During this phase, the door between the subject and partner rooms was open, giving the subject access to the reward after a correct choice. When they chose the control symbol, there was no reward. Following either choice, after 6–8 seconds of a white screen, the next trial was presented.

Training was completed when subjects chose the giving symbol on 17/20 trials (significantly higher than chance performance, binomial test) within one session, not necessarily in consecutive trials, and readily moved to the partner enclosure to receive the reward (S1 Data). They had to reach this criterion in two sessions, first whilst still using the clicker and a second time with no clicker as secondary reinforcement. Additionally, they had to make their choices within a time limit of 15 seconds after the symbols appeared on the screen for each trial. This time limit was set as in the test and control sessions, the software terminated a session after 15 seconds of no input. Therefore we had to be sure that the subjects would work for themselves within this time limit before test and control sessions began.

### Testing procedure

Each subject participated in one session of each of the five conditions. Two of these conditions were test conditions, one with the in-group partner and the other test with the out-group partner. During these test sessions, the partner was in the partner room (S1 Fig). The three other conditions were control conditions, with one social facilitation control condition per partner and one non-social control condition. During the social facilitation control conditions, the partner was outside of the touch screen room in a fenced area at the same distance from the touch screen room as the partner room (S1 Fig), so they could not access the food but they were still in visual, olfactory and auditory contact with the subject. Finally, there was no partner at all in the non-social control condition.

Conditions were presented in a semi-counterbalanced order across dyads, in such a way that the two conditions involving the same partner were performed one after the other (test and social facilitation control). Furthermore, the sequence of the two testing conditions was the same with the two partners (e.g. if the subject started with the test condition with the in-group partner, he also started with the test condition with the out-group partner). Finally, the non-social control condition was randomly positioned before, between or after the in-group and the out-group conditions. Within a test/control trial, subjects were given 15 seconds to choose a symbol. The session ended when the subject twice refused to choose a symbol within the allotted time limit. This marked the extinction point of the subject for that condition and was used as the measure of prosociality. The point at which subjects chose to end the session was compared across the conditions. A session lasted for a maximum of 80 trials.

A motivation session was conducted in between conditions. These motivation sessions were the same as the final training session, where the door between the subject and partner rooms was open and subjects were required to choose the giving symbol on 17/20 trials before moving on to the next session. Since the experimental design was measuring the extinction of a previously rewarded behaviour, whereby subjects did not obtain any food in the test or control conditions, the purpose of these motivation sessions was to bring the subjects back up to a baseline level of performance before the next condition, with the aim of reducing any impact of previous testing sessions on the following condition.

Immediately at the end of each session, subjects could complete six trials for themselves. During these trials the partner (when present) remained in the same position but the experimenter moved in order to reward the subject in the touch screen room. Therefore, if subjects worked for themselves on these self-rewarding trials, it is clear that their score on the prior session reflects their level of prosociality, rather than other social factors such as discomfort or distraction from the partner. In these six self-rewarding trials, all subjects of both species performed every trial.

### Statistical analysis

Firstly, we ran a correlation between the number of trials performed and the number of giving trials (Pearson's correlation test, r = 0.972; p < 0.001). The significant correlation means that when subjects performed a trial, they usually touched the giving symbol. This suggests that they were not making use of the control symbol as a strategy to avoid giving food and that they simply did not touch the screen when they did not want to give food. The number of giving trials was therefore used as our measure of prosociality as it reflects specifically how many food items were delivered by the subjects.

Due to the high amount of individual variation in the responses (S1 Data), we calculated difference scores by subtracting the number of giving trials in the non-social control from the number of trials performed in the other conditions per subject (see S1 Data). All statistical analyses were thus carried out using these difference scores. To assess whether the dogs and wolves were prosocial in our study, we compared the differences scores of giving trials performed by the subjects via generalized linear mixed model analyses (GLMM), correcting for overdispersion by using the glmmADMB package and function in R version 3.2.2 [47]. Initially all conditions were included in a model to investigate whether an interaction between species and condition existed. The fixed explanatory variables in the models were the species, the condition and the session (i.e. the order of the five conditions), as well as the interactions between condition and session and condition and species. Since the dogs showed no effect of condition, the subsequent models were run only on the wolf data. The first model compared the three control conditions (social facilitation control with each of the partners and the non-social control). The second model compared the in-group test with the in-group control. The final model compared the out-group test with the out-group control. The fixed effect was condition and for all models subject was included as a random effect.

The animals’ care is in accordance with institutional and national guidelines. The research was discussed and approved by the institutional ethics committee at the University of Veterinary Medicine, Vienna in accordance with GSP guidelines and national legislation (10/08/97/2014). The individual in this manuscript has given written informed consent (as outlined in PLOS consent form) to publish these case details.

## Supporting information

S1 Fig Detailed diagram of the testing set-up. (JPG)

S2 Fig The touch screen apparatus. (JPG)

S3 Fig The pool of three stimuli used in the test. Each subject was randomly assigned two of these; one as the ‘giving’ and one as the ‘control’ symbol.
(TIF)

S4 Fig Mean number of giving by wolves and dogs in the three control conditions. (TIF)

S1 Data Raw data file. (XLSX)
