# Supplementary figures and images for: Wolves, but not dogs, are prosocial in a touch screen task
Source: PLoS One. 2019 May 1;14(5):e0215444. doi: 10.1371/journal.pone.0215444 (PMC6493736; doi:10.1371/journal.pone.0215444)

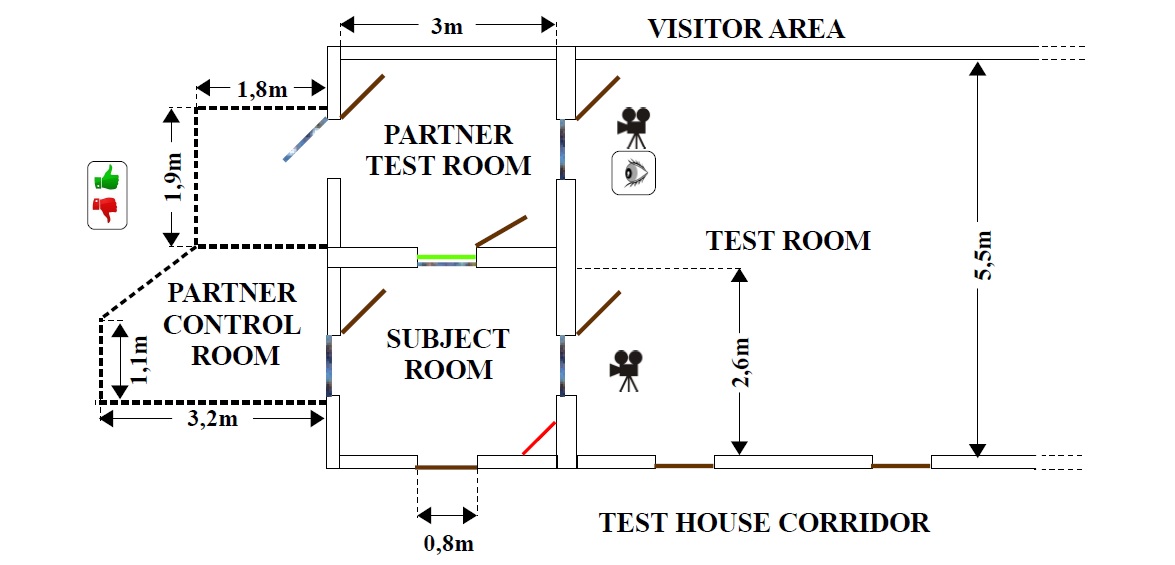

Supplement: S1 Fig — (JPG) [file pone.0215444.s001.jpg]

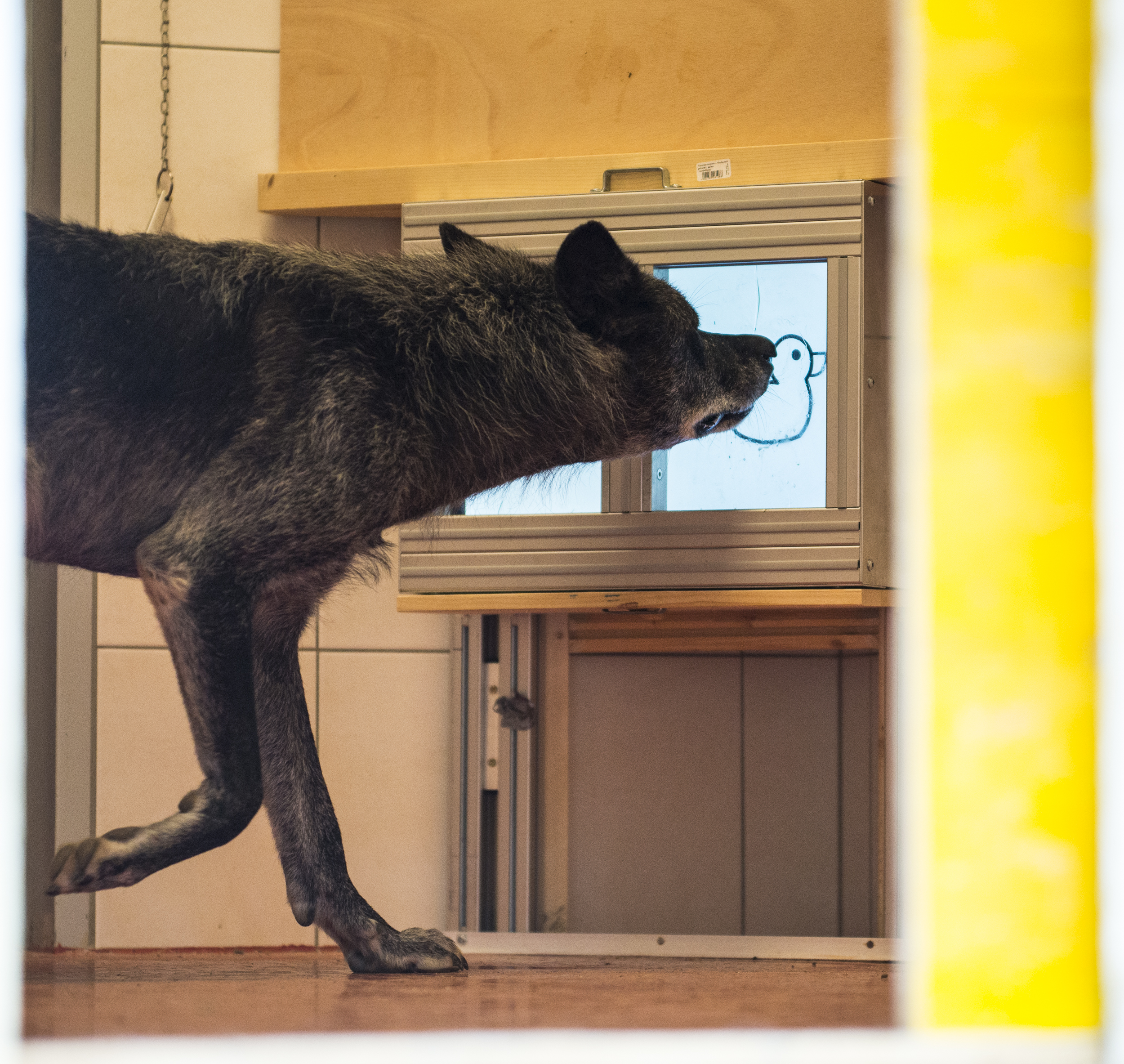

Supplement: S2 Fig — (JPG) [file pone.0215444.s002.jpg]

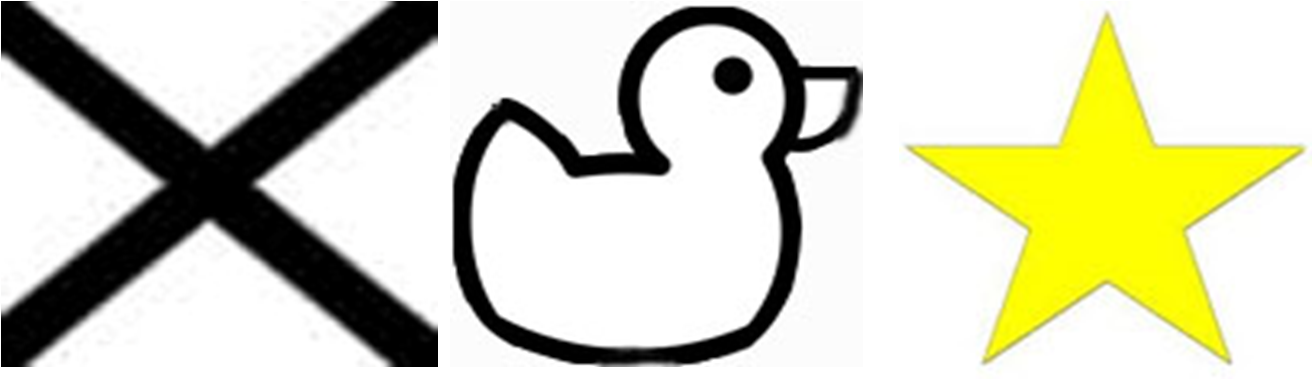

Supplement: S3 Fig — Each subject was randomly assigned two of these; one as the ‘giving’ and one as the ‘control’ symbol. (TIF) [file pone.0215444.s003.tif]

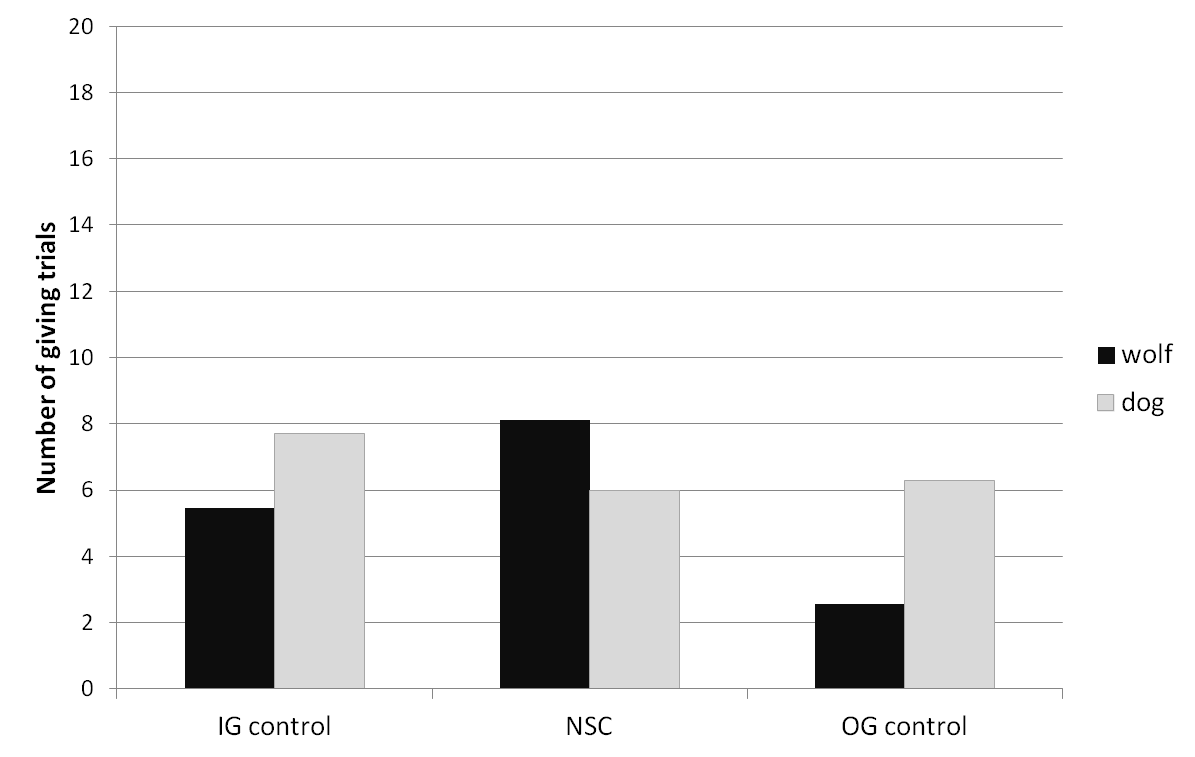

Supplement: S4 Fig — (TIF) [file pone.0215444.s004.tif]
